# Toward earlier identification and preventative intervention in schizophrenia: evidence from the London Child Health and Development Study

**DOI:** 10.1007/s00127-015-1151-x

**Published:** 2015-12-15

**Authors:** Kristin R. Laurens, Alexis E. Cullen

**Affiliations:** Department of Forensic and Neurodevelopmental Sciences, Institute of Psychiatry, Psychology & Neuroscience, King’s College London, London, UK; Health Service and Population Research Department, Institute of Psychiatry, Psychology & Neuroscience, King’s College London, London, UK; Research Unit for Schizophrenia Epidemiology, School of Psychiatry, University of New South Wales, Sydney, Australia; Schizophrenia Research Institute, Sydney, Australia

**Keywords:** Psychosis, High-risk, Developmental psychopathology, Psychotic-like experiences, Adolescence

## Abstract

**Purpose:**

The London Child Health and Development Study (CHADS) is a prospective, longitudinal investigation of children, sampled from the general community aged 9–11 years and assessed biennially, who present premorbid risk markers for schizophrenia. The study aims to characterise developmental trajectories of psychological, cognitive, and biological functioning in at-risk children and identify potential targets for early preventative intervention. This review summarises CHADS findings, discusses these in the context of recent theory regarding aetiology and prevention of schizophrenia, and highlights challenges to be addressed with future research.

**Methods:**

We review (1) epidemiological information on the prevalence and correlates of developmental antecedents of schizophrenia in the general child population, (2) evidence of psychosocial, cognitive, and biological dysfunctions in at-risk children presenting multiple antecedents of schizophrenia and at-risk children with a family history of schizophrenia, and (3) related findings from an associated sample of help-seeking children receiving intervention.

**Results:**

Community-based screening of 9–11-year olds identified ~9 % with a triad of antecedents of schizophrenia [including psychotic-like experiences (PLEs)] who are putatively at-risk of psychosis; these children reported greater exposure and responsivity to stressors, impairments in general intelligence and specific cognitive functions, brain structure and function abnormalities, and neuromotor dysfunction. Preliminary evidence suggests distressing PLEs are a viable target for cognitive-behavioural intervention in at-risk children.

**Conclusions:**

Intervention in early, premorbid phases of illness might alleviate current difficulties and avert future schizophrenia using benign treatments. The CHADS programme has identified several markers that may index early pathophysiology and constitute potential targets for preventative intervention.

## Introduction

Over the past two decades, considerable research and clinical effort has been invested in devising methods of early detection and intervention for psychosis, with the aims of delaying, ameliorating, and ultimately preventing, illness onset [1]. This work has focussed predominantly on the identification and treatment of symptomatic, help-seeking individuals purportedly experiencing the prodromal phase of illness that immediately precedes the onset of frank psychosis, typically during later adolescence or early adulthood. Within 2–3 years of presentation, a third of these “clinically high risk (CHR)^1^” individuals transition to psychotic illness [2]; a substantial proportion continue to experience persistent psychopathology, marked psychosocial impairment, and compromised quality of life; and only a third experience clinical remission [3]. This trajectory of persisting or worsening functional disability for the majority underscores a need for earlier intervention. That is, preventing psychosis and associated adverse outcomes might be more effective if we could identify at-risk individuals in the premorbid phase of illness, prior to the advent of the significant disability that characterises the prodrome, and without restriction to those accessing health services. Motivated by this aspiration, the London Child Health and Development Study (CHADS) was designed as a prospective, longitudinal, observational investigation of a cohort of children sampled from the general community at age 9–11 years, and enriched with individuals putatively at high risk for developing schizophrenia and the spectrum disorders (SSD). The primary goal of the study was to characterise developmental trajectories of at-risk individuals through adolescence and into young adulthood, with the aim of determining markers of evolving disease that, in the longer term, might be targeted with early, preventative interventions.

Established methods for the identification of individuals potentially experiencing the psychosis prodrome are based on clinical assessment of help-seeking individuals.^2^ Our research required a new method for prospectively identifying at-risk children in the premorbid phase of illness via screening of the general population. Although family history of schizophrenia represents the most established of risk markers for the illness (~10–15 % of individuals with a first-degree relative with schizophrenia develop the disorder), a lack of family history for the majority with schizophrenia [6] renders this a limited means of identifying at-risk individuals in the population. During 2004–2005, we therefore piloted a novel method of screening community samples in the United Kingdom (UK), using self- and caregiver-report questionnaires [7], to identify putatively at-risk children who presented multiple, replicated, developmental antecedents of schizophrenia. These antecedents, identified in previous prospective longitudinal investigations as distinguishing children who later developed SSD from those who did not [8–10], are thought to represent early manifestations of the disease pathology (compared to “risk factors”, which may be conceptualised as more passive markers of increased risk). As these antecedents are not specific for SSD relative to other adult psychiatric disorders (albeit their association with SSD is typically of greater relative magnitude), we reasoned that a combination of antecedents would identify putatively at-risk children with greater sensitivity and specificity than any single antecedent. Our brief questionnaire provided a cost-effective means of screening large numbers of children in the general population for the antecedents within a primary school setting, with sampling restricted to children aged 9 years and older so as to obtain reliable and valid self-reports. As some of the strongest and/or most replicated antecedents of SSD identified in systematic reviews [8–10] are not amenable to accurate assessment via questionnaire (e.g. obstetric complications, premorbid intellectual [IQ] deficits), our questionnaire incorporated antecedents that were strongly associated with later schizophrenia and suited to questionnaire assessment.^3^ We additionally assessed family history of mental health problems (including SSD).

Within the CHADS programme, children completing questionnaire screening provided a community sample (cross-sectional data) from which several longitudinal cohorts were convened, including (1) an unselected community sample of children who have been assessed sporadically using questionnaires and interviews, and (2) a selected sample of children who have completed detailed laboratory-based assessments biennially. This review summarises CHADS findings published to date, including epidemiological findings (from the community cohorts) and psychological, cognitive, and neurobiological findings (from the selected longitudinal cohort), as well as findings from an associated sample of help-seeking children receiving intervention, discusses these findings in the context of recent theory regarding aetiology and prevention of SSD, and outlines challenges remaining for future research.

## Methods

Table 1 summarises the community and selected cohorts incorporated within the CHADS programme and their associated publications.

**Table 1:** Summary of CHADS programme cohort characteristics (pilot, community, selected, and associated intervention cohorts) and associated publications

CHADS cohort	Sample characteristics	Associated publications
1. Pilot study sample (2004–2005)	548 children aged 9–11 years (mean age 10.6 years; 54.7 % female) and 264 primary caregivers, comprising: 129 children and caregivers who completed questionnaires via General Practitioner surgeries (21.6 % of 597 children enrolled at collaborating surgeries) 421 children (97.7 % of children eligible) who completed questionnaires in school class and 136 caregivers (32.3 %)	[7]
2a. Community sample (screening)	7966 children completing questionnaires anonymously in school class (94.6 % of children eligible; mean age 10.4 years; 49.2 % female) and 1504 primary caregivers (18.9 %)	[9, 17, 85] (subset of sample); [15]
2b. Longitudinal community sample	670 children and caregivers (mean age 10.3 years; 53.4 % female)—the subset of 799 families who provided identities and contact information at screening and whose contact information remained valid at first reassessment approximately 2 years later	[27, 28, 73]
3. Longitudinal selected sample (biennial assessments)	111 children and caregivers, comprising: 46 TD; 33 ASz; 26 FHx; and 6 children meeting both ASz and FHx criteria^a^ Biennial assessments completed at ages 9–12 years (baseline); 11–14 years (follow-up 1); 13–16 years (follow-up 2); and 17–18 years (follow-up 3) (Note: a further 39 children with alternative illness risk profiles, including bipolar disorder, were recruited in insufficient numbers to provide viable groups for examination)	[30, 34, 35, 39, 43, 48, 52, 53, 55, 56]
4. CHADS-associated intervention samples	Clinical case series: four children from the community sample who completed a new, manualised cognitive behavioural intervention for children presenting psychotic-like experiences and emotional distress	[74]
	Coping with unusual experiences for children study (CUES): doi 10.1186/ISRCTN13766770 (Lead: Dr. S. Jolley)—for children aged 8–14 years presenting to Child and Adolescent Mental Health Services with unusual experiences and emotional distress	[75–78]
	Coping with unusual experiences for 12–18 (CUES+): doi 10.1186/ISRCTN21802136 (Lead: Dr. S. Jolley)—for users of adolescent community mental health services (aged 12–18 years) who report distressing unusual experiences	

*CHADS* London Child Health and Development Study, *TD* typically developing children, *ASz* children presenting a triad of developmental antecedents of schizophrenia, *FHx* children with a family history of schizophrenia/schizoaffective disorder

^a^Includes eight FHx children recruited via contact with patients receiving treatment in the local health service rather than via school screening

### Community (cross-sectional and longitudinal) cohorts

Our sampling and recruitment strategies are detailed elsewhere [7, 9]. Briefly, during 2005–2010, children aged 9–11 years were sampled from 73 collaborating primary schools within Greater London.^4^ Children (*n* = 7966; 95 % of eligible children) completed questionnaires independently and anonymously in class, with items read aloud by a researcher. Caregivers completed corresponding questionnaires (matched by code) at home and returned them via reply-paid mail (*n* = 1504); this constituted the cross-sectional community sample [Table 1, “2a. Community sample (screening)”].

Caregivers were invited to provide child and caregiver identities and contact information, indicating their willingness to consider further research participation (*n* = 799 families). A longitudinal community sub-cohort (Table 1, “2b. Longitudinal community sample”) was derived from the screening sample and assessed for representativeness,^5^ with sampling weights applied in analyses where appropriate.

### Selected longitudinal cohort

From the screening questionnaires, a triad of antecedents of schizophrenia (ASz) were defined to include: (1) child-reported subclinical psychotic symptoms [or psychotic-like experiences (PLEs)], (2) child-reported emotional symptoms and/or caregiver-reported behavioural or social problems, and (3) caregiver-reported delays or abnormalities in speech and/or motor development [7, 9]. Three groups of children were recruited from the community screening sample and followed with biennial assessments that measured psychological, cognitive, and biological features previously shown to be characteristic features of adults with schizophrenia, forming a longitudinal selected sample of 111 children (Table 1, “3. Longitudinal selected sample (biennial assessments)”). The groups incorporated two high risk groups, namely children presenting the antecedent triad (ASz group) and children with a family history (in first- or second-degree relatives) of schizophrenia or schizoaffective disorder (FHx group), as well as a group of low risk, typically developing children who presented no antecedents or family history of schizophrenia (TD group). Table 2 details the measures included in the five assessment phases conducted to date in this selected cohort.^6^

**Table 2:** Summary of the assessments completed with the longitudinal selected sample at each assessment phase

Domain	Assessment method (informant)	Assessment instrument	Assessment phase				
			SCR	BL	FU1	FU2	FU3^a^
Psychopathology							
Social, emotional, and behavioural problems	Questionnaire (P, C, T)	Strengths and Difficulties Questionnaire (SDQ [11, 12, 86]): psychopathology scales (emotional symptoms, peer relationship problems, conduct problems, hyperactivity–inattention), prosocial behaviour scale, and supplement assessing impact of psychopathology	✓	✓	✓^+^	✓	✓
DSM-IV diagnoses and symptoms (including psychotic symptoms)	Interview (P, C)	Kiddie Schedule for Affective Disorders and Schizophrenia—Present and Lifetime version (K-SADS-PL) [87] [primary caregiver: full schedule; child: anxiety and psychotic symptoms (screening and supplement) questions only]	–	✓	–	–	–
	Questionnaire (P, C, & T)	Achenbach System of Empirically Based Assessment (ASEBA [88]): Child Behavior Checklist (CBCL), Youth Self-Report (YSR), Teacher Report Form (TRF)	–	✓	✓	✓	✓
Anxiety symptoms	Questionnaire (C)	Revised Child Manifest Anxiety Scale, second edition (RCMAS-2 [89])	–	✓	✓	✓	–
Depressive symptoms	Questionnaire (C)	Beck Depression Inventory for Youth (BDI-Y [90])	–	✓	✓	✓	–
Antisocial traits	Questionnaire (P, C)	Antisocial Process Screening Device [91]	–	✓	✓	✓	–
Autistic symptoms	Questionnaire (P)	Social Communication Questionnaire—lifetime version [92]	–	✓	–	–	–
Psychotic-like experiences	Questionnaire (P, C)	Psychotic-Like Experiences (PLE) Questionnaire [7, 15]: items assessing hallucination- and delusion-like experiences, and associated impact	✓	✓	✓^+^	✓	✓
Prodromal symptoms of psychosis	Questionnaire (C)	Prodromal Questionnaire [93]	–	–	–	✓	✓
Psychotic symptoms	Interview (C)	Comprehensive Assessment of At-Risk Mental State (CAARMS [94])	–	–	–	–	✓
Personality	Questionnaire (P)	Big Five Inventory [95] adapted for Children	–	–	–	✓	–
Global functioning	Interview (C)	Global Assessment of Functioning scale (GAF [96])	–	✓	–	–	✓
Medical and developmental history							
Developmental delays	Questionnaire (P)	Items assessing delays/problems in the attainment of speech or motor milestones [7]	✓	–	–	–	–
Medical and psychiatric history	Interview (C)	Family Interview for Genetic Studies (FIGS [97]) to identify known medical and psychiatric conditions in the child and other family members	–	✓	✓	✓	–
Relationships assessment							
Parenting practices	Questionnaire (P, C)	Alabama Parenting Questionnaire [98]	–	✓	✓	✓	–
Family functioning	Questionnaire (P)	McMaster Family Assessment Device [99]	–	–	–	✓	–
Expressed emotion	Standardised rating (P)	Expressed emotion rating scale (coded from video recording) [100, 101]	–	✓	✓	✓	–
Child experiences							
Alcohol and drug use	Questionnaire (C)	Adapted version of the Edinburgh Study of Youth Transitions and Crime alcohol and drug use questionnaires [102]	–	✓	✓	✓	✓
Daily hassles and life events	Questionnaire (C)	Adapted version of a daily school-related hassles and negative life events questionnaire [103]	–	✓	✓	✓	–
Threatening life events	Questionnaire (C)	List of Threatening Events (LTE [104])	–	–	–	–	✓
Perceived stress	Questionnaire (C)	Perceived Stress Scale (PSS [105])	–	–	–	–	✓
Childhood trauma	Questionnaire (C)	Childhood Trauma Questionnaire (CTQ [106])	–	–	–	–	✓
Victimisation	Interview (C)	Juvenile Victimization Questionnaire 2nd revision (JVQ-R2 [107])—sections on victimisation by peers and siblings, internet/mobile harassment, and discriminatory attacks	–	–	–	–	✓
Self-harm	Questionnaire (C)	Item assessing self-harm in the past 12 months [108]	–	–	–	–	✓
Socio-environmental indices							
Sociodemographic variables	Questionnaire (P, C)	Items assessing child’s sex, date of birth, and (by caregiver-report only) child’s self-ascribed ethnicity, parents’ dates and places of birth, child and family history of mental health problems, and whether child had ever lived outside London	✓	–	–	–	–
Ethnicity	Questionnaire (P)	Office of Population Censuses and Surveys method of self-ascription (2001 census)	–	✓	✓	✓	–
Migration history	Questionnaire (P)	Date of migration and country of origin	–	✓	✓	✓	–
Home, school, and community supports	Questionnaire (C)	Middle Development Index items [109]	–	✓	✓	✓	–
Sociodemographic context	Questionnaire (P)	UK Medical Research Council Sociodemographic Schedule on housing and living (migration) circumstances; religious engagement	–	✓	✓	✓	–
Socio-economic Status	Questionnaire (P)	National Statistics Socio-Economic Classification (NS-SEC [110]); household income; educational attainment	–	✓	✓	✓	–
Financial income	Questionnaire (C)	Items assessing income sources and total income per week	–	–	–	–	✓
Biological indices							
DNA/mRNA	Buccal sample (C)	Collected at the research session and in home environment	–	✓	✓	✓	✓
	Blood sample (C)	Collected at the research session	–	✓	✓	✓	✓
Cortisol	Saliva samples (C); hair sample (C)	Home collection of multiple saliva samples throughout the day at FU2 [43]; hair sample collected in the research session at FU3	–	–	✓	–	✓
Pubertal status	Questionnaire (P, C)	Pubertal Development Scale [111]	–	✓	✓	✓	–
Neuromotor function							
Laterality/handedness	Questionnaire (C)	Annett Hand Preference Questionnaire [112], plus items from the Edinburgh Handedness Inventory [113] and Coren’s Lateral Preference Scale [114]	–	✓	✓	✓	–
Gross and fine motor skills	Standardised test (C)	Purdue Pegboard [115]	–	✓	✓	✓	–
Involuntary dyskinetic movements	Standardised rating (C)	Dyskinesia Identification System Condensed User Scale [116] (coded from video recording)	–	✓	–	–	–
Brain structure and function							
General intelligence	Standardised test (C)	Wechsler Abbreviated Scale of Intelligence (WASI [117])	–	✓	✓	✓	✓
Scholastic achievement	Standardised test (C)	Wechsler Individual Achievement Test 2nd UK edition (WIAT [118]): word reading, numerical operations, and spelling subtests	–	✓	✓	✓	–
Processing speed	Standardised test (C)	Symbol Digit Modality Test [119]	–	–	–	✓	–
Memory	Standardised test (C)	Wide Range Assessment of Memory and Learning 2nd edition (WRAML2 [120]): verbal learning and memory, visual learning and memory, verbal delayed recall, recognition (verbal and nonverbal), and working memory subtests	–	✓	✓	✓	–
Executive function	Standardised test (C)	Delis-Kaplan Executive Function System (D-KEFS [121]): verbal fluency, colour–word interference (Stroop), and tower test subtests	–	✓	✓	✓	–
Facial emotion recognition	Computer task (C)	Penn Emotion Discrimination Task (EmoDiff40) [122]	–	✓	✓	✓	–
Facial emotion discrimination	Computer task (C)	Emotion Recognition Test–40 Faces version (ER-40) [123]	–	✓	✓	✓	–
Brain structure	Magnetic Resonance Imaging (C)	Spoiled Gradient Recalled acquisition (Magnetization Prepared Rapid Acquisition Gradient Echo—additional scan completed in a subset only)	–	✓	✓	✓	–
Mismatch negativity	Computer task (C)	Duration deviant passive auditory oddball task: ERP recordings [55]	–	✓	✓	✓	–
Working memory	Computer task (C)	Spatial N-Back task: functional MRI (variant of [124])	–	✓	✓	✓	–
Attention	Computer task (C)	Auditory novelty oddball task: ERP recordings and functional MRI (variant of [125])	–	✓	✓	✓	–
Error-related processing	Computer task (C)	Go/No-Go task: ERP recordings and functional MRI [53]	–	✓	✓	✓	–
Response inhibition	Computer task (C)	Stop task: ERP recordings and functional MRI (variant of [126])	–	✓	✓	✓	–
Service use, support, and quality of life							
Service utilisation	Questionnaire (P, C)	Services Assessment for Children and Adolescents [127]	–	–	✓^+^	–	✓^+^
Resource access	Questionnaire (P, C)	Resource Generator—UK, expert advice subscale [128]	–	–	✓^+^	–	✓^+^
Social cohesion	Questionnaire (P, C)	Items assessing neighbourhood social cohesion [129]	–	–	✓^+^	–	✓^+^
Peer interaction	Questionnaire (C)	Child Health and Illness Profile [130] peer interaction and satisfaction scale	–	–	–	–	✓^+^
Mental health literacy	Questionnaire (C)	Self-report questionnaires on two vignettes depicting persons with (1) depression and (2) psychosis. Assesses recognition of the disorder, intended help-seeking, beliefs about interventions and prevention, stigmatising attitudes, and exposure to mental disorders [131]	–	–	–	–	✓^+^
Self-identification as having a mental illness	Questionnaire (C)	Self-Identification as Having a Mental Illness Scale (SELF-I) assessing perceived need for professional help and appraisal of problem as mental illness [132] adapted for young people	–	–	–	–	✓^+^
Stigma							
Public stigma	Questionnaire (P, C)	Reported and Intended Behaviour Scale [133]	–	–	✓^+^	–	✓^+^
Stigma coping	Questionnaire (C)	Items assessing cognitive appraisal of mental health stigma [134]	–	–	–	–	✓^+^

Assessment phase—*SCR* screening assessment (age 9–11 years; data collected during 2005–2010 in 7966 children screened with questionnaires at primary school), *BL* baseline assessment (age 9–12 years), *FU1* follow-up assessment 1 at approximately 24-months post-baseline (age 11–14 years), *FU2* follow-up assessment 2 at approximately 48-months post-baseline (age 13–16 years), *FU3* follow-up assessment 3 at approximately 72-months post-baseline (age 17–18 years; ^a^self-report information only collected)

*P* primary caregiver, *C* child, *T* teacher

^+^Indicates measures collected in the longitudinal community cohort in addition to the longitudinal selected cohort

### Related intervention cohorts

We additionally summarise findings obtained to date from CHADS-associated intervention research with help-seeking children (Table 1, “4. CHADS-associated intervention samples”); that is, children identified through mental health services and not community screening.

## Results

### Epidemiological findings

Data from our CHADS cross-sectional and longitudinal community sampling have provided important epidemiological information concerning the prevalence and correlates of developmental antecedents of schizophrenia in the general child population, particularly PLEs.

#### Developmental antecedents of SSD

**Fig. 1:**
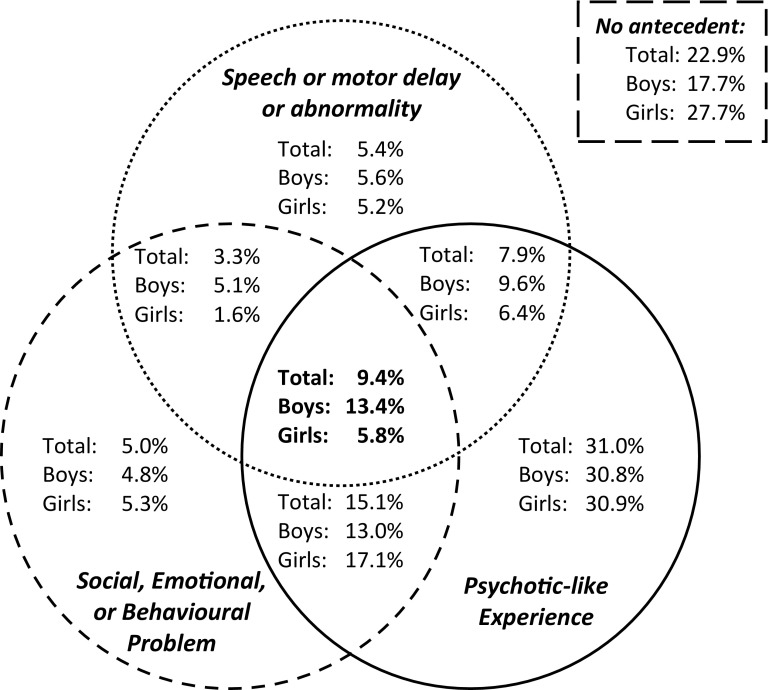
Venn diagram indicating the percentages of children displaying antecedents within each domain of the triad (overlapping segments indicate children who displayed multiple antecedents); based on data from 1504 child–caregiver dyads who completed screening questionnaires

Consistent with the elevated incidence of schizophrenia in the London community from which our sample was drawn [16], more than three quarters of children aged 9–11 years experienced at least one of the antecedents within the triad (Fig. 1), with just under a tenth (9.4 %) presenting all three. Caregiver reports of a family history of schizophrenia on the screening questionnaires were similarly elevated (3.4 % of children). The antecedent triad was significantly more prevalent in males relative to females (Fig. 1), and more common among children of African-Caribbean [9, 17] and black African [9] ethnicity relative to white British children, mimicking the increased incidence of schizophrenia [18, 19] in these ethnic minority groups in the UK. Conversely, children of south Asian ethnicity were less likely relative to the white British population to present the antecedent triad [9], providing a potential opportunity to identify protective, as well as risk, processes that might be operating prior to illness onset in schizophrenia.

#### Childhood PLEs

An early contribution from the CHADS programme was the development of a nine-item self-report questionnaire assessing a range of delusion- and hallucination-like experiences for use by children aged 9-11 years [7], comprising an adaption and extension of items from the Diagnostic Interview Schedule for Children [20]. Almost two-thirds of children reported at least one PLE, implying that these experiences may be part of a spectrum of normative childhood experience in middle childhood [15]. Recent meta-analyses indicate that auditory hallucinations are more prevalent among children aged 9–12 years (median prevalence: 17 % [21]) than in the adolescent (7.5 %) or adult (5–8 % [22]) general population, with questionnaire measures typically eliciting higher rates than clinical interviews [23]. Discrepancy in our rates of self- and caregiver-reported childhood PLEs (66.0 vs. 9.7 %; also reported by others [24, 25]) implies that children do not necessarily report these phenomena to adults, or that they interpret their experiences differently than do caregivers. Whilst the majority of children reporting PLEs will not go on to develop a SSD in adulthood, for many children, these experiences are not benign; more than a third (40.9 %) reported distress or functional impairment associated with these experiences, particularly those presenting the antecedent triad (68.1 % [9]). In children, similarly to the adult population [26], the PLE items load on a construct which is correlated with, but dissociable from, the constructs underpinning internalising and externalising problems [15]. Two items assessing hallucination-like experiences appeared most suited to identifying children with vulnerability to psychotic illness in the general population [15], with such items previously also showing strong criterion validity for psychotic symptoms elicited by diagnostic interview among 11–13-year olds [24].

#### Persistence of PLEs

Meta-analytic data approximate that 75–90 % of developmental psychotic experiences are transitory and relatively benign, but that these experiences may persist to become clinically relevant, depending on the degree of environmental risk to which the person is additionally exposed [22]. Our longitudinal data indicated that two in five children (39 %) who reported PLEs at baseline continued to report these experiences approximately 2 years later, in adolescence [27]. Persistent PLEs from childhood (9–11 years) were associated with internalising and externalising psychopathology in adolescence [27], implying that interventions targeting persistent PLEs might not only alleviate risk for later schizophrenia, but more immediately, reduce common adolescent psychopathology.

#### Trajectories of psychopathology

Though PLEs and internalising and externalising psychopathology in middle childhood all constitute replicated antecedents of schizophrenia, our data indicate that internalising and externalising psychopathology experienced only during childhood is not associated with increased risk for PLEs in adolescence, whereas psychopathology that persists from childhood into adolescence or is incident in adolescence confers increased risk for later PLEs [28]. This work suggests a need for further investigation into timely targeted interventions designed to prevent progression from early symptom development to full disorder.

### Psychological, cognitive, and neurobiological findings

Detailed, laboratory-based assessments have been conducted biennially in the selected sample to determine the extent to which hallmark disturbances of schizophrenia are present already during the early (pre-prodromal) at-risk phase of illness; findings to date are summarised in Table 3.

**Table 3:** Summary of psychopathological, cognitive, neurobiological, and HPA axis abnormalities observed among children presenting antecedents of schizophrenia (ASz) and a family history of illness (FHx) compared with the pattern of abnormalities typically observed among adults with established psychosis

Marker	Adult psychosis vs. HC	ASz vs. TD children	FHx vs. TD children
Psychopathology and stress responsivity			
Social withdrawal^b^	↑	↑	↑
Psychosocial stress exposure^d^	↑	↑	↑
Psychosocial stress reactivity^d^	↑	↑	↑
Cognitive impairments^c^			
General intelligence (IQ)	↓	↓	↓
Scholastic achievement	↓	↓	↓
Verbal memory	↓	↓	↓
Visual memory	↓	**–**	**–**
Working memory (verbal)	↓	↓	↓
Executive function (verbal fluency)	↓	↓	↓
Executive function (inhibition)	↓	↓	↓
Facial emotional processing ability	↓	↓	Not examined
Neurobiological abnormalities			
Grey matter volume (left STG/MTG and right MTG)^a^	↓	↑ ↓	Not examined
White matter volume (left SLF, ILF, and OR)^a^	↓	↑	Not examined
Error-related negativity ERP component amplitude^a^	↓	↓	Not examined
Mismatch negativity ERP component amplitude^a^	↓	↑	Not examined
Dyskinetic movement abnormalities^a^	↑	↑	Not examined
HPA axis dysfunction			
Diurnal cortisol^d^	↑	–	–
Cortisol awakening response^d^	↓	–	↓
Pituitary volume^d^	↑ ↓	–	–

*HC* healthy control, *TD* typically developing children, ↓ decreased in psychosis/high-risk group relative to HC/TD group; ↑ Increased in psychosis/high-risk group relative to HC/TD group, – no difference between psychosis/high-risk group and HC/TD group, *STG* superior temporal gyrus, *MTG* medial temporal gyrus, *SLF* superior longitudinal fasciculus, *ILF* inferior longitudinal fasciculus, *OR* optic radiation, *ERP* event-related potential, *HPA* hypothalamic–pituitary–adrenal

Encompassing CHADS assessments completed at age ^a^ 9–12 years, ^b^ 9–14 years, ^c^ 9–16 years, and ^d^ 11–14 years

#### Psychopathology and stress responsivity

Prospective longitudinal studies of both general population [10] and familial high-risk [29] cohorts indicate psychopathology during childhood and adolescence in individuals who later develop SSD. We investigated social withdrawal in children with different vulnerability profiles for the disorder, namely, children at putatively elevated symptomatic risk of schizophrenia (ASz) and those at elevated genetic risk due to a family history of illness (FHx). Both ASz and FHx children aged 9–14 years presented significantly elevated levels of parent-reported social withdrawal relative to their TD peers, though the magnitude of the effect was greater among ASz children [30]; thus, social withdrawal may be a more prominent feature among children at elevated symptomatic risk.

With respect to their response to psychosocial stressors (e.g. major life events, childhood trauma, and milder daily hassles), which have been shown to contribute to the development and maintenance of psychosis in retrospective and prospective studies [31–33], FHx and ASz children aged 11–14 years reported greater exposure to negative life events and daily hassles, respectively, compared to TD children, and were more distressed by these experiences [34]. Thus, while both groups were more responsive to psychosocial stressors, ASz and FHx children may be susceptible to different stressors. Relative to TD children [35], both risk groups experienced higher rates of physical punishment (thought to be on a continuum with childhood maltreatment, a replicated risk factor for SSD [36]).

#### Cognitive impairment

In light of meta-analytic data indicating premorbid IQ impairment in children who later develop schizophrenia [37], with low IQ increasing the risk of later schizophrenia in a dose–response fashion [38], we investigated trajectories of neurocognitive function in children with different vulnerability profiles. In initial cross-sectional analyses conducted in ASz and TD groups only, we observed impairments in general intelligence, verbal memory, working memory, and executive function among ASz children aged 9–12 years [39] which were less pervasive and smaller in magnitude than those characterising adults with schizophrenia [40], but similar to those observed among CHR individuals [41, 42]. In subsequent analyses performed on a larger, partially overlapping sample, ASz children performed at a level intermediate to FHx children with high familial loading (≥1 first-degree or ≥2 second-degree relatives) and FHx children with low familial loading (one affected second-degree relative only) across a range of neurocognitive subtests [43]. Preliminary analysis of longitudinal data collected across three biennial assessments spanning 9–16 years indicates different patterns of cognitive development through adolescence in ASz and FHx children relative to TD children. ASz and FHx children exhibited stable deficits in IQ, scholastic achievement, verbal working memory, and specific domains of executive function, but greater gains in verbal memory relative to their TD peers [44], with relatively more subtle differences apparent between the high risk groups (as has been indicated also in meta-analysis of older samples of CHR youth relative to youth with family history [45]). Our findings highlight the uneven pace of development of different cognitive abilities throughout adolescence in at-risk youth, which might reflect divergence in the rate of maturation in some brain areas among at-risk youth compared to their TD peers.

The cognitive impairments characterising ASz children also extend to social cognition. Consistent with the impaired ability of individuals with schizophrenia [46] and CHR youth [47] to recognise facial emotions, ASz children (9–15 years) showed moderate deficits in facial emotion recognition, particularly sad and angry expressions [48]. Future analyses will examine whether these facial emotion processing deficits also characterise FHx children; previous investigations of older youth (13–25 years) indicate that such impairments are more prominent among CHR individuals than those with family history of schizophrenia [49].

#### Neurobiological abnormalities

Antipsychotic medication and neurodegenerative processes associated with disease progression potentially confound neurobiological studies of adults with established schizophrenia, and only the latter are overcome in studies of CHR youth (a substantial proportion of whom also receive psychotropic treatment [50]). Our work examining medication-naive at-risk children thus offers important insights into the aetiology of neurobiological abnormalities underlying schizophrenia.

We have shown that a subset of the structural brain abnormalities associated with schizophrenia (typically, widespread volume decreases, but also increases, encompassing the frontal and temporal lobes, medial temporal regions, anterior cingulate, insula, and thalamus [51]) precede the prodromal phase of illness. By age 9–12 years, ASz children present significant grey matter volume reduction in the right middle temporal gyrus, but significant volume increase in the left superior and middle temporal gyri relative to TD children [52]. ASz children also show increased white matter volume in the left inferior parietal lobe, occipital lobe, and superior temporal gyrus, corresponding to parts of the superior longitudinal fasciculus, inferior longitudinal fasciculus, and optic radiation. Whilst these abnormalities contrast with temporal lobe volume reductions typically reported in schizophrenia, our findings imply that changes in this region may be among the first structural brain abnormalities to emerge. Our longitudinal data will allow us to track further structural changes through adolescence to illness onset.

Abnormalities of brain function that characterise adults with schizophrenia, as indexed by event-related potentials (ERP), are present also in ASz children. We have investigated two well-defined ERP components at 9–12 years: error-related negativity (ERN), a brain potential elicited following detection of an error, and auditory mismatch negativity (MMN), an ERP component that reflects an automatic attentive process detecting discrepancy between an incoming sound and the memory trace of preceding sounds. Similar to patients with schizophrenia, ASz children showed reduced amplitude of the ERN component [53]. It is proposed that the ERN is generated to an error following a dopamine-mediated negative reinforcement learning signal sent from the basal ganglia to the anterior cingulate cortex [54]; thus, reduced ERN in schizophrenia (and ASz children) might reflect disruption of these dopamine pathways. In contrast with the decreased MMN amplitude typically observed in schizophrenia, however, ASz children were characterised by increased MMN amplitude relative to TD children [55], indicating the need for longitudinal data to establish the developmental trajectory of this component in at-risk children.

We have further demonstrated that involuntary dyskinetic movements, thought to index the abnormal striatal dopamine levels that characterise individuals with schizophrenia, are more frequent among ASz children aged 9–12 years compared to TD children [56]. These abnormalities (rated blindly using videotapes) were observed in the facial regions (e.g. tics, grimacing) and upper body (e.g. shoulder/hip torsion, finger or wrist extensions) and are similar to those reported among children who later develop schizophrenia [57, 58] and adolescents with schizotypal personality disorder [59].

#### Hypothalamic–pituitary–adrenal (HPA) axis dysfunction

Our study of medication-naïve, non-help-seeking children has also allowed us to investigate whether abnormalities within the HPA axis (the primary system involved in coordinating the physiological response to stress), which have been observed among first-episode psychosis patients and CHR youth [60–63], also characterise earlier stages of illness. It is hypothesised that, among individuals at increased vulnerability for psychosis, HPA axis hyperactivity (triggered by psychosocial stress) elicits elevated cortisol levels, which in turn contribute to the clinical features of psychosis by augmenting dopamine activity [64, 65]. However, HPA abnormalities reported among first-episode and CHR patients [including elevated daytime cortisol levels, a blunted cortisol awakening response (CAR), and pituitary volume enlargements] might simply be a consequence of the stress associated with emerging illness.

By age 11–14 years, FHx children (but not ASz children) showed a blunted CAR [43] that was not explained by experiences of psychosocial stressors and was more prominent among FHx children with a first-degree relative with schizophrenia than among FHx with an affected second-degree relative. In contrast to hypotheses, neither ASz nor FHx children were characterised by higher diurnal cortisol levels. Nonetheless, among both FHx and ASz children, abnormal cortisol levels (i.e. higher diurnal cortisol levels and greater blunting of the CAR) were associated with poorer memory and executive function, possibly reflecting underlying dysfunction in the brain regions which mediate both HPA axis function and these cognitive functions [43]. Neither ASz nor FHx children were characterised by pituitary volume enlargements [35], which contrasts with those identified among individuals with first-episode psychosis [66–69] and in some (but not all) studies of older individuals at-risk for psychosis [69, 70]. Among FHx children only, pituitary volume was negatively associated with current distress relating to negative life events and exposure to physical punishment [35], implying that psychosocial stressors may contribute to pituitary volume changes among those with family history. Overall, our findings tentatively suggest that the blunted CAR might be an early (possibly genetically mediated) marker of psychosis vulnerability, while HPA axis hyperactivity (as indexed by elevated daytime cortisol levels and enlarged pituitary volume) might emerge closer to disease onset.

### Intervention findings

Many children and adolescents experiencing mental health difficulties do not receive appropriate professional care [71, 72]. Data from the CHADS longitudinal community cohort have highlighted the central role of caregivers’ attitudes and experiences on young people’s service use (in health and education settings), particularly the influence of caregivers’ perceptions of stigma and their own service use history [73]. Targeting stigma among caregivers may be a key strategy in bridging the gap between young people’s need and service use.

We have developed a cognitive behavioural intervention for children aged 9–14 years who experience PLEs and emotional problems which is designed to reduce emotional symptoms, improve coping and resilience, and help children manage distressing PLEs [74]. In a pilot with four children, child and therapist satisfaction with the programme was high, and emotional problems and PLE frequency and impact all decreased during intervention. A randomised controlled evaluation of the intervention with clinically referred samples of children is underway (Table 1, “4. CHADS-associated intervention samples”). Distressing PLEs are common in these children. In pre-treatment data, negative life events, emotional symptoms, cognitive biases (e.g. probabilistic reasoning and jumping to conclusions biases), and negative schematic beliefs about self and others independently contributed to PLE severity [75, 76]. Further, negative schematic beliefs mediated the relationship between experiences of bullying and PLEs [77]. These psychosocial and cognitive processes show different associations with PLE content (e.g. paranoia, hallucinations) and dimensional attributes (e.g. frequency, impact) [78] and provide potential targets for psychological intervention in children with PLEs.

## Discussion

The CHADS programme, encompassing complementary community and selected cohorts, has yielded findings that may aid efforts to delineate the aetiological processes underlying SSD, and inform strategies to identify and treat at-risk individuals earlier than is currently feasible. Building on the work of early prospective longitudinal studies of both general population and familial high-risk cohorts (which were typically restricted to examination of psychopathological features), we have identified several neurobiological markers that may index early, premorbid pathophysiology on a developmental trajectory to SSD. By means of our large, unselected sample (from an ethnically diverse, urban population), we have contributed epidemiological findings demonstrating that the prevalence of the triad of schizophrenia antecedents is elevated among males and specific ethnic groups, and that PLEs in particular can be distressing and persistent for some children, depending on co-occurrence of internalising and externalising symptoms or other antecedents of schizophrenia. Furthermore, in a clinically referred sample of children, we have shown that levels of distress and severity associated with PLEs are related to psychological and cognitive constructs that may be amenable to cognitive-behavioural interventions.

### Scientific and theoretical advancements

#### Aetiological theories

Our findings align with the recent sociodevelopmental-cognitive model of schizophrenia [79] that integrates dopaminergic, neurodevelopmental, sociodevelopmental, and cognitive theories. Whilst it has long been suspected that schizophrenia is preceded by abnormal neurodevelopment commencing in early life, prospective studies utilising magnetic resonance imaging and ERP technology have been lacking. Our work has provided preliminary evidence that several structural [52] and functional brain abnormalities [53, 55] associated with schizophrenia are evident in at-risk children by age 9–12 years. Moreover, increased dyskinetic movements [56] and reduced amplitude of the ERN brain potential [53] in ASz children hint at early abnormalities in dopamine regulation. In line with the cognitive component of the model, our work in clinically referred children has indicated a number of cognitive biases associated with PLEs [75, 76]. We have identified a number of potential social adversities which might contribute to such biases (e.g. physical punishment [35], negative life events, and daily hassles [34]) that are more prevalent and elicit greater distress in at-risk children than typically developing children. Whilst we have found no evidence as yet that these stressful experiences elicit HPA axis abnormalities in ASz and FHx children [35, 43], developmental changes may render the HPA axis more susceptible to environmental stressors. Thus, many of the components within the sociodevelopmental-cognitive model of schizophrenia [79] that are hypothesised to contribute to the development of schizophrenia are detectable in at-risk children, but further work is required to integrate these findings for testing.

#### Genetically and symptomatically driven risk markers

The CHADS findings augment those of previous longitudinal studies, commencing in the 1950s, which prospectively followed offspring of parents with schizophrenia [29]. We offer evidence confirming neurocognitive impairments and social withdrawal in young relatives of individuals with schizophrenia [80–82], and new findings regarding experiences of psychosocial stress and HPA axis function in FHx children (which, until now, have been studied only in adult relatives who may no longer be ‘at-risk’ for the disorder). By characterising development of both ASz and FHx children, we may identify risk markers that are primarily genetically mediated (and may be shared by non-symptomatic relatives), and others present only in individuals displaying the antecedent phenotype [83]. The latter offers the prospect of identifying candidate mechanisms associated with emerging illness. Future investigations might benefit from incorporating at-risk groups identified by alternative means (e.g. youth with a family history of bipolar disorder or those with 22q11.2 deletion syndrome).

#### Timing and trajectories

To date, we have investigated only a subset of the potential markers that may be affected in at-risk children, and while many of these markers have shown abnormalities that are similar to those observed in adults with schizophrenia and CHR youth, there are notable exceptions. For example, ASz children were characterised by increased amplitude of the MMN potential [55], and relative increases in grey and white matter in the temporal lobes [52]; it is possible that these patterns may reverse as the brain undergoes maturation in adolescence. Our investigations revealed no evidence of anticipated abnormalities such as elevated diurnal cortisol [43] or pituitary volume enlargement [35] among either ASz or FHx children, implying that HPA axis hyperactivity may emerge more proximally to illness onset. Having identified potential premorbid markers of illness (albeit these must be shown to be robust with replication in other samples), an important next step will be to determine whether these markers vary with symptom fluctuation and are responsive to intervention. Studies that assess changes in both biological and psychological markers in response to intervention are needed.

### Implications

The psychosocial, cognitive, and neurobiological features that we have found to characterise ASz and FHx children might potentially be used to enhance the accuracy with which individuals at-risk of SSD can be identified in the general population and/or via relatives with SSD. We anticipate that, instead of SSD, some putatively at-risk children will develop other psychiatric disorders, whilst others will develop no disorder. Longitudinal follow-up of the cohort is needed to determine the specificity and sensitivity with which the antecedent triad and associated psychopathological markers distinguish between these outcomes. From a clinical perspective, our findings have already informed the development of a new psychological intervention for children aged 8–14 years which targets current difficulties experienced by the children (e.g. distressing PLEs and emotional symptoms [74]). We hope that such intervention might also avert more serious mental health problems in the future.

Research into the early identification and prevention of SSD has focussed predominantly on youth in late adolescence or early adulthood who present features consistent with the prodromal phase of illness immediately preceding psychosis onset, with little consideration given to vulnerable children or younger adolescents in an earlier phase of illness. Our research demonstrates that this group, who may present different clinical and biological features and treatment needs to those meeting CHR criteria [84], should not be overlooked. Indeed, children presenting multiple antecedents of schizophrenia present a range of social, psychological, cognitive, and biological abnormalities characteristic of adults with schizophrenia, though they are (at present) less marked and diffuse, thereby providing a potential window for early intervention. Our new method of screening community samples to identify children displaying multiple antecedents of schizophrenia (who are putatively in the premorbid illness phase) offers a tool to facilitate research, and might, with refinements informed by ongoing research, complement established methods for identifying CHR youth in the later, prodromal phase of illness. Further research across the CHADS cohorts is underway to: (1) characterise developmental changes through adolescence and into young adulthood that might constitute viable targets for early preventative interventions, (2) identify neurobiological, neurocognitive, and psychopathological changes that might signal imminent risk of transition to psychosis, and (3) develop novel, innovative interventions that might alter the course of illness in vulnerable individuals. Through these research endeavours, we hope ultimately to improve outcomes for individuals who may be on the trajectory to this devastating disorder.

## Footnotes

1 The CHR state is also known as the “at-risk mental state” (ARMS) and “ultra-high-risk” (UHR) state [1].

2 Established methods (reviewed in [1]) for the identification of individuals in this prodromal phase include the complementary UHR [4] and basic symptoms [5] criteria assessed via clinical interview, with the latter possibly identifying an earlier phase of the prodromal state than UHR criteria.

3 Items assessed psychotic-like experiences (child- and caregiver-report), internalising and externalising psychopathology (emotional symptoms, peer relationship problems, conduct problems, and hyperactivity–inattention; child- and caregiver-report using the Strengths and Difficulties Questionnaire [11, 12]), and delays or abnormalities in speech and/or motor milestone development (caregiver-report). Additional items assessed family history of mental health problems including SSD (caregiver-report), and demographic items (child- and caregiver-report). See Table 2 (screening phase) for further detail.

4 Collaborating schools spanned the range of socio-economic disadvantage represented in London schools (as indexed by eligibility of children to receive free school meals; with oversampling of deprived inner-London communities) and included state and religious schools of variable enrolment size. Eighty percent of children attended school within the inner-city London Boroughs of Lambeth* (35.1 %), Southwark* (22.1 %), Lewisham* (18.6 %), Tower Hamlets* (2.6 %), and Westminster (1.6 %), with the remaining fifth sampled from the outer London Boroughs of Croydon (8.5 %), Bromley (7.0 %), Brent* (3.2 %), and Harrow (1.4 %). Boroughs indicated by * are among the most deprived 10 % of all English local authorities according to the Index of Multiple Deprivation 2010 [13] and the lowest scoring 10 % on the Local Index of Child Well-Being [14]. School-level data obtained from the Office for National Statistics at the time of sampling indexed the socio-economic status and ethnic diversity of the sample: On average, 31.2 % of children in participating schools were eligible to receive free school meals (range 1.9–62.2 %; cf. Greater London average 25.3 %, and England average 15.8 %); 24.9 % of children were of white British ethnicity (range 0–80.8 %; cf. Greater London average 37.1 %, and England average 80.3 %), with the remainder of black (45.2 %), Asian (11.4 %), other white (9.0 %), or other (9.5 %) ethnicities.

5 Representative indices (by child self-report) include basic demographic information (age, sex), PLEs [7, 15], and emotional symptoms, peer relationship problems, conduct problems, and hyperactivity–inattention [11, 12]. Few differences between the cross-sectional and longitudinal community samples are notable on these measures (see primary publications and [9]).

6 Assessment phases in the longitudinal selected sample include: screening (9–11 years), baseline (9–12 years), and first (11–14 years), second (13–16 years), and third follow-up (17–18 years). Measures that were assessed more widely, in the longitudinal community sample, are indicated in Table 2 using the symbol “+”. The initial questionnaire assessment which provided cross-sectional data in the community sample (from which the longitudinal community sample and longitudinal selected samples were derived) is summarised under the screening phase.
